# Identification of Functional Single Nucleotide Polymorphisms in Porcine *HSD17B14* Gene Associated with Estrus Behavior Difference between Large White and Mi Gilts

**DOI:** 10.3390/biom10111545

**Published:** 2020-11-12

**Authors:** Siyuan Gao, Ruixin Tao, Xian Tong, Qinglei Xu, Jing Zhao, Yanli Guo, Allan P. Schinckel, Bo Zhou

**Affiliations:** 1 College of Animal Science and Technology, Nanjing Agricultural University, Nanjing 210095, China; 2018105082@njau.edu.cn (S.G.); 2018105011@njau.edu.cn (R.T.); 2017105081@njau.edu.cn (X.T.); 2019205004@njau.edu.cn (Q.X.); 2019105039@njau.edu.cn (J.Z.); 2019105040@njau.edu.cn (Y.G.); 2 Department of Animal Sciences, Purdue University, West Lafayette, IN 47907-2054, USA; aschinck@purdue.edu

**Keywords:** 17β-hydroxysteroid dehydrogenase type 14, apoptosis, estrogen, estrus behavior, granulosa cells, pigs, SNPs

## Abstract

Steroid hormone levels are associated with estrous behavior, which affects timely mating and reproductive efficiency in pigs. 17β-hydroxysteroid dehydrogenase type 14 (*HSD17B14*) modulates steroid synthesis and metabolism. To identify the functional single nucleotide polymorphisms (SNPs) in the porcine *HSD17B14* gene, ear tissues from Large White and Mi gilts were collected to extract genomic DNA. Variable lengths of truncated promoter of *HSD17B14* gene were used to determine the promoter activity by a dual luciferase reporter system. The vector *HSD17B14*^Phe^ or *HSD17B14*^Val^ was transfected into porcine granulosa cells (GCs). The core promoter region was identified between −72 bp and −218 bp. Six of seven SNPs had significant differences of allele frequency between Large White and Mi gilts. The plasmids with the wild genotype AA of rs329427898 maintained a smaller fraction of promoter activity compared with the plasmids with the mutant genotype GG, while the plasmids with wild the genotype TT of rs319864566 had a greater promoter activity than the plasmids with the mutant genotype CC. A missense mutation (Phe73Val) caused changes in the structural dynamics and function of the *HSD17B14* protein. The highly expressed *HSD17B14*^Val^ degraded less estradiol into estrone, while the relatively lowly expressed *HSD17B14*^Phe^ degraded more estradiol into estrone, suggesting the protein activity of *HSD17B14*^Phe^ was greater than that of *HSD17B14*^Val^. Moreover, the *HSD17B14*^Phe^ group has a greater apoptosis rate of porcine GCs. The *HSD17B14* gene could been used as a candidate molecular marker for estrus behavior in pigs.

## 1. Introduction

The beginning of the estrous cycle occurs at the onset of estrus when the female animal is sexually receptive (known as in heat) and is followed by the ovulation of follicles [1]. In sows and gilts, estrus signs include a swollen red vulva, riding behavior between pen mates, seeking a boar, standing in response to the presence of a boar or a back-pressure test [2]. However, the proportion of gilts that do not express estrus behaviors has increased over the past few decades [3,4], which has hindered estrus detection and increased the number of non-productive days of sows [2,5]. Thus, exploring the molecular regulatory mechanism of estrous behavior expression in sows and gilts could contribute to resolving the problem of anestrus and improving pig productivity [6].

Positive correlations have been found between serum estrogen concentrations and the intensity of estrus behavior of gilts [7,8]. Reproductive hormone secretion and physiological changes of gilts during the estrus cycle are regulated by multiple molecular interactions in reproductive related tissues, such as the hypothalamus, pituitary, ovary, oviduct, and endometrium [9]. The synthesis of ovarian estrogen is regulated by luteinizing hormone (LH) and follicle-stimulating hormone (FSH) in the theca cell and granulosa cells (GCs) [10]. During the growth and development of follicles, LH stimulates the theca to secrete testosterone, and then, under the stimulation of FSH, GCs convert testosterone to estradiol, namely, this process is termed “double cell dual-sex hormone effect mode” [10,11].

As a family of multifunctional enzymes, 17β-Hydroxysteroid dehydrogenases (17β-HSDs) modulate the synthesis and metabolism of steroids by reducing 17-ketosteroids or oxidizing 17β-hydroxysteroid to produce NAD(P)H or NAD(P)+ as a cofactor [12]. 17β-HSDs control the formation and conversion of several reproductive hormones, such as androgen and estrogen [13]. Estrogens and androgens, such as estradiol (E_2_), 5-androstene-3β, 17β-diol (5-diol), and testosterone (T), have been identified as substrates of this enzyme [14,15]. It converts estradiol (E_2_) into estrone (E_1_) both in vivo and in vitro [14]. Moreover, the cytosolic localization of *HSD17B14* could allow “control by access” by limiting exogenous and endogenous active estrogen (estradiol) reaching ERs (estrogen receptors) located in the nucleus [14,16,17]. Estradiol, as a kind of estrogen, maintains greater estrogenic activity than estrone. Previous studies have demonstrated that the characteristics of estrus were associated with the level of plasma estrogen in gilts [8,9].

Previous studies have shown that Chinese indigenous pig breeds, such as Meishan, Erhualian, and Mi pig breeds, have superior reproductive performance and estrus expression traits than European pig breeds [18,19,20,21]. Gilts of these Chinese pig breeds reach puberty at an earlier age [22], express behavioral estrus for longer, and have slightly shorter estrus cycles [18]. Compared with Large White gilts, Mi gilts have a longer duration of standing reflex, greater scores of vulva reddening and greater serum estradiol-17β concentration [23].

Our previous study found that the expression level of the *HSD17B14* gene was greater in Mi gilts than in Large White gilts [24]. We hypothesized that the difference in estrogen levels between Mi and Large White gilts during estrus may be related to the expression and function of the *HSD17B14* gene. The objective of this study was to identify the functional single nucleotide polymorphisms (SNPs) of the *HSD17B14* gene associated with differences in estrus behavior observed between the Large White and Mi gilts. Several SNPs in the porcine *HSD17B14* gene have been identified with significant differences of genotype frequency between Large White and Mi gilts. The effects of these SNPs on the expression of the porcine *HSD17B14* gene were investigated in vitro. Furthermore, we verified the effects of a missense mutation in the fifth exon of *HSD17B14* on expression, function and apoptosis in porcine ovarian GCs.

## 2. Materials and Methods

### 2.1. Animals and Sample Collection

This study was approved by the Animal Care and Use Committee of Nanjing Agricultural University (SYXK2017-0007). A total of 100 gilts (50 Large White and 50 Mi gilts) were randomly selected in Yongkang Agricultural Science and Technology Co., Ltd. in Changzhou, Jiangsu, China. Estrus detection was carefully performed twice daily (at 7:00 and 15:00) at pro-estrus. Expression of estrus was defined as standing reflex, and reddening and swelling of the vulva. Inspections to evaluate standing reflex, and reddening and swelling of the vulva were made on gilts individually by an experienced technician using a standardized routine [3]. A sexual mature boar was introduced into each pen for at least 15 min daily to ensure adequate stimulation. Standing reflex was assessed by the back-pressure test. Reddening and swelling of the vulva was measured visually by a trained person. The identification of estrus behavior includes the color of the vulva, the color of vaginal mucus, the amount of mucus, the viscosity of mucus, vocalization of gilts, and climb across behavior. When a standing reflex occurred, the expression of estrus was scored (0: no; 1: weak; 2: strong) via the use of scoring criteria [23]. After the first scoring, Large White and Mi gilts with scores of 2 points were selected. The specific scoring criteria have been used in previous studies [24]. According to the scoring standard of estrus characteristics, at the onset of standing reflex in the second estrus cycle, a 5 mL sample of blood was collected from the anterior vena cava of gilts at the 10th day of the estrus cycle. Blood samples were centrifuged at 3000 rpm for 10 min, immediately after collection blood samples of Large white and Mi gilts were collected for estrogen determination [23]. Before the third estrus cycle, 6 Large white gilts and 6 Mi gilts were selected. The estrus cycles were about 21 d in length, at the first day of the third estrous, the day the gilts were at the point of onset of exhibiting the standing reflex, three Large White and three Mi gilts were sacrificed humanely with anesthesia. At the 10th day of estrous cycle, three Large White and three Mi gilts were sacrificed in the same way. The ovary, spleen, ileum, kidney, stomach, brain, eye muscle, and liver were dissected, and all samples were collected from the gilts [24]. All samples were immediately frozen in liquid nitrogen and stored at −80 °C until RNA was isolated. We have provided the estrus behavior of the Large White and Mi gilts in the Supplementary Table S6.

### 2.2. Cell Culture, Cell Transfection, and Luciferase Assays

Transfections in human renal epithelial cell-293T cells (ATCC^®^ACS-4004™) were performed using Lipofectamine 2000 (Invitrogen, Carlsbad, CA, USA). Cells were plated in 12-well plates. On the following day, the *HSD17B14* promoter-luciferase plasmid was co-transfected with plasmid pRL-TK (the herpes simplex virus thymidine kinase promoter fused upstream to the Renilla luciferase gene, which was used as an internal control; Promega, Madison, WI, USA) into the 293T cells. Controls were the pGL3-basic and pGL3-control luciferase reporter gene vector instead of the *HSD17B14* promoter luciferase plasmids. After 24 h, cells were harvested with luciferase assay buffer (Promega, Madison, WI, USA). The cell lysates were assayed for luciferase activity using the Promega Dual Luciferase Assay system. For porcine GC culture, ovaries were obtained from seven-month old unstimulated commercial replacement Large White gilts at a local slaughterhouse. The ovaries were quickly washed twice with 75% ethanol and physiologic saline, and a syringe was used to extract the follicular fluid from a follicle with a diameter of 3–5 mm [25]. GCs were cultured with Dulbecco’s minimum essential medium/nutrient F-12 (DMEM/F-12, Gibco, Gaithersburg, MD, USA) supplied with 15% fetal bovine serum (FBS, Gibco, Gaithersburg, MD, USA) at 37 °C in a humidified atmosphere of 5% CO_2_ for 48 h.

### 2.3. RNA Isolation and RT-qPCR

Total RNA of ovarian tissue was extracted from six Large White gilts and six Mi gilts using TRIzol (Invitrogen, Carlsbad, CA, USA) according to the manufacturer’s instructions. The purity of RNA was determined with a NanoPhotometer^®^ Spectrophotometer (IMPLEN, Westlake Village, CA, USA) at 260/280 nm. To quantify *HSD17B14* messenger RNA (mRNA) levels, total RNA was reverse-transcribed using the HiScript III RT SuperMix for qPCR (Vazyme Biotech, Nanjing, China). RT-qPCR was performed on a QuanuStudio 5 using SYBR Green Master Mix (Vazyme Biotech, Nanjing, China). Relative expression levels were calculated by using the 2^−ΔΔCt^ method [26]. Coding gene expression levels were normalized to the expression of *GAPDH*. PCR reactions were performed in triplicate and the primers used are shown in Supplementary Table S2.

### 2.4. Promoter Prediction of the Porcine HSD17B14 Gene

The promoter region of the porcine *HSD17B14* gene was predicted by Promoter 2.0 (http://www.cbs.dtu.dk/services/Promoter/) [27] and neural network promoter prediction (https://www.fruitfly.org/seq_tools/promoter.html) [28]. Putative transcriptional binding start sites were predicted by PROMO (http://alggen.lsi.upc.es/cgi-bin/promo_v3/promo/promoinit.cgi?dirDB=TF_8.3) [29,30]. Methylation sites were predicted using Meth-Primer 2.0 (http://www.urogene.org/methprimer/) [31]. MicroRNA binding sites in the 3-untranslated region (UTR) region of porcine *HSD17B14* gene were predicted by Target Scan 7.2 (http://www.targetscan.org/vert_72/) [32]. Protein structure was predicted using PSIpred-MEMSAT3 (http://bioinf.cs.ucl.ac.uk/psipred/psiform.html) [33].

### 2.5. Potential SNP Identification

Genomic DNA of 100 gilts (50 Large White and 50 Mi gilts) was extracted from ear tissue by a standard phenol/chloroform method. The specific primers (Supplementary Table S1) were designed using Primer5 to amplify 5’-UTR, 5 exons and 3’-UTR of the porcine *HSD17B14* gene. PCR reactions were performed using 1.1 × T3 Super PCR Mix (TsingKe, Nanjing, China). The amplified PCR products were sequenced, and multiple sequence splices and alignments were performed to analyze the SNPs in Large White and Mi gilts using the software DNAMAN 8.0 (https://www.lynnon.com/index.html) and Chromas (v 2.6.4, Technelysium Pty Ltd., South Brisbane, Australia).

### 2.6. Plasmid Construction

The promoter region of the porcine *HSD17B14* gene was amplified by PCR using Taq DNA polymerase (Takara, Dalian, China). Subsequently, plasmids containing variable lengths of truncated the porcine *HSD17B14* promoter were individually amplified using different forward primers and a common reverse primer (Supplementary Table S2). For plasmid ligation, the forward primers and reverse primer contained Sac I and Hind III recognition sequences, respectively (*HSD17B14-P1*: −2057/+254, *HSD17B14-P2*: −1273/+254; *HSD17B14-P3*: −972/+254; *HSD17B14-P4*: −218/+254; *HSD17B14-P5*: −949/−701; *HSD17B14-P6*: −720/+395). The amplified fragments were then inserted into the multiple cloning site of the pGL3-basic vector to generate luciferase reporter plasmids. Specific regions containing SNP rs342163057 were amplified using *HSD17B14*-P5 primers and SNPs rs329427898 and rs319864566 were amplified using *HSD17B14*-P6 primers. The forward and reverse primers contained Sac I and Hind III recognition sequences.

For the missense mutation rs342747498 (T > G), *HSD17B14*^Phe^ and *HSD17B14*^Val^ overexpression plasmids were built, porcine *HSD17B14* CDS (Supplementary Table S1) was amplified and double-digested with Xba I and Hind III, and then cloned into the pcDNA3.1(+) vector (Invitrogen). All plasmids were sequenced to confirm proper insertion prior to transfection experiments.

### 2.7. Western Blotting

Cell protein lysates were collected using 200 μL radio immunoprecipitation assay (RIPA) buffer with 1% Phenylmethylsulfonyl fluoride (PMSF) (*v*/*v*) and total protein extracts were separated on 4–20% SDS–PAGE gels (Genscript, Biotechnology, Piscataway, NJ, USA) and blotted onto polyvinylidene fluoride (PVDF). After membranes were blocked with 5% bovine albumin (BSA) in TBST (Tris-Hcl, NaCl, tween20) buffer at 4 °C for 2 h, the membranes were incubated overnight with the following primary antibodies: Immunoreactive proteins were detected with a rabbit polyclonal antibody for *HSD17B14* (1:2000; CUSABIO, Houston, TX, USA) and a rabbit polyclonal antibody for β-actin (1:5000; Affinit, Nanjing, China). The appropriate secondary antibodies were used by anti-rabbit (1:10,000; Affinit) and chemiluminescence was detected by an Image LAS-4000 system. The band density was analyzed using ImageJ software.

### 2.8. E_1_ and E_2_ Detection

The cell culture medium (about 2 mL) was collected 48 h after transfection with pcDNA3.1, *HSD17B14*^Phe^, and *HSD17B14*^Val^. According to the manufacturer’s instructions, constructing a standard curve for the standards provided in the E_1_ and E_2_ kit. The standard curve of Porcine E_1_ and E_2_ ELISA Kit was *y* = 0.0015*x* + 0.0595 (R^2^ = 0.9964 > 0.95) and *y* = 0.0012*x* + 0.043 (R^2^ = 0.9942 > 0.95), respectively. The absorbance value (OD value) of each well was measured at a 450 nm wavelength in accordance with the operation manual. E_1_ and E_2_ levels under different experimental conditions were measured using a Porcine E_1_ ELISA Kit and an E_2_ ELISA Kit (MEIMIAN, Beijing, China).

### 2.9. Cell Apoptosis Analysis

GCs were harvested at 48 h after transfection and fluorescence-activated cell sorting (FACS) was performed to measure apoptosis using a cell counting machine (Becton Dickinson, Franklin Lakes, NJ, USA). The experiments were conducted using an Annexin V-FITC/PI Apoptosis Detection Kit (Vazyme, Nanjing, China) according to the manufacturer’s protocol. In total, 10,000 cells were detected and apoptosis rate was calculated. The data were analyzed using the FlowJo v7.6 software (Stanford University, Stanford, CA, USA).

### 2.10. Statistical Analyses

Linkage Disequilibrium (LD) and haplotype distributions of SNPs were analyzed using the expectation maximization algorithm in Haploview 4.2 (https://www.broadinstitute.org/haploview/downloads). Statistical analyses were performed using IBM SPSS Statistics for Windows (version 25.0, Chicago, IL, USA). The differences of genotypic frequency between breeds were analyzed using the chi-square test. An unpaired two-sided student’s *t*-test and one-way analysis of variance were used to evaluate the significance of statistics. Means were reported as means ± standard error of the mean (SEM). Tests were considered significant at *p* < 0.05.

## 3. Results

### 3.1. Tissue Expression Profile of HSD17B14 Gene in Gilts

**Figure 1:**
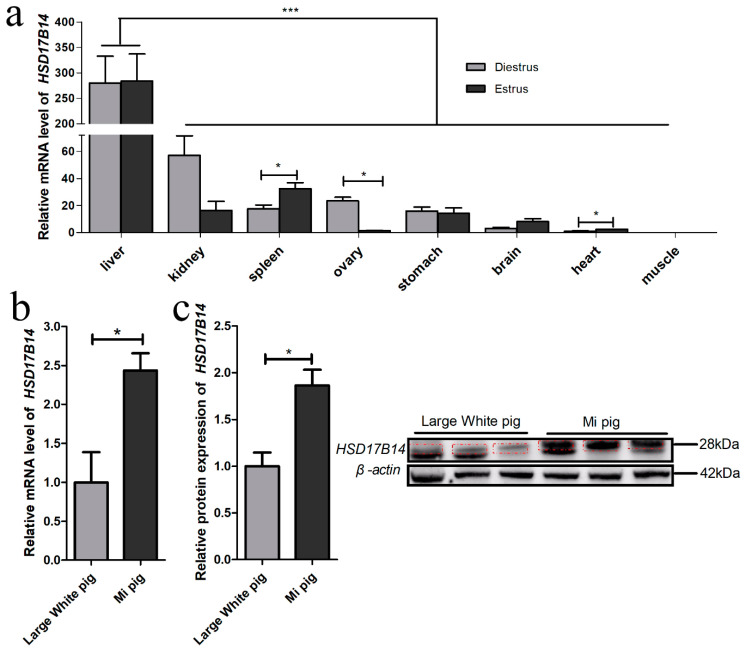
Expression pattern of porcine *HSD17B14* gene. (**a**) Expression characteristics of porcine *HSD17B14* gene at diestrus and estrus. (**b**) RT-qPCR of Large White and Mi pigs in diestrus. (**c**) Western blot of Large White and Mi pigs in diestrus, the red dotted lines were the purpose strip. Each experiment was repeated three times and the results are represented as means ± S.E.M. * stands for *p* < 0.05 and *** stands for *p* < 0.001 (*t*-test).

The mRNA expression levels of the *HSD17B14* gene were determined by RT-qPCR in the spleen, liver, ovary, kidney, brain, stomach, heart and muscle (*Longissimus dorsi*) of gilts in diestrous and estrus (Figure 1a). The porcine *HSD17B14* gene had a greater expression level in the liver than the other seven tissues (*p* < 0.05) (Figure 1a). However, there was a significant difference between the period of diestrus and estrus, only present for the ovary (*p* = 0.0013), heart (*p* = 0.0134) and spleen (*p* = 0.0435) (Figure 1a). The *HSD17B14* gene expression level in the ovary was greater in Mi gilts than in Large White gilts (*p* = 0.0326) (Figure 1b). At the same time, Western blotting of their ovarian tissues showed that the protein level of *HSD17B14* was greater in Mi pigs than in Large White pigs (*p* = 0.0177) (Figure 1c).

### 3.2. Promoter Prediction of HSD17B14 Porcine Gene

Two promoter regions (−1757/−1668 and −574/−524), and three transcription initiation sites (−1757, −1257, and −457) of the porcine *HSD17B14* gene were identified by Promoter 2.0 and neural network promoter prediction. Three CpG island signals (−1293/−1082, −666/−561, and −39/+198) were identified using Meth-Primer 2.0. The 5’-UTR region of porcine *HSD17B14* gene promoter harbors potential binding sites for multiple transcription factors including ENKTF-1, C/EBPbeta, NF-1, STAT4, p53, Sp1, FOXP3, AR, E2F-1, Elk-1, NFI/CTF, AP-1, c-Fos (Supplementary Table S3).

### 3.3. Genotypic Frequencies of HSD17B14 Gene in Large White and Mi Pigs and Linkage Disequilibrium (LD) Analyses

**Figure 2:**
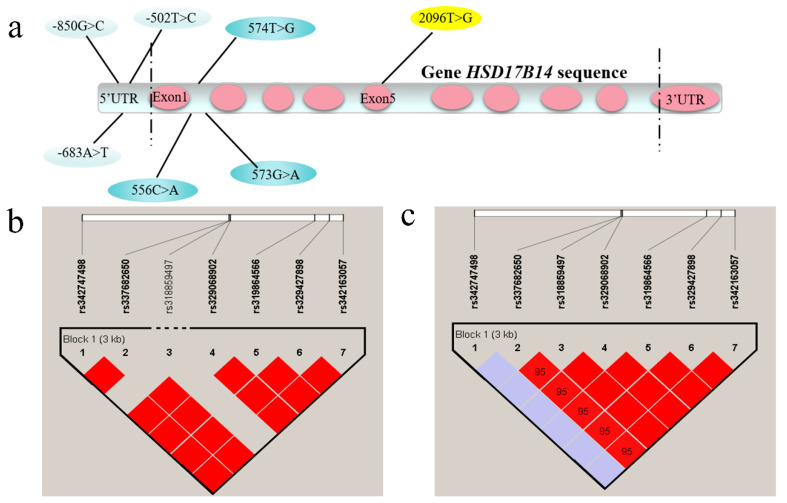
The linkage disequilibrium (LD) analysis of 7 SNPs in the porcine *HSD17B14* gene. (**a**) The position of seven SNPs in the porcine *HSD17B14* gene. (**b**) LD analysis of the seven potential SNPs in Large White gilts. (**c**) LD analysis of the seven potential SNPs in Mi gilts.

Three SNPs, rs342163057 (−850G > C), rs329427898 (−683A > G), and rs319864566 (−502T > C), were identified in the 5’-UTR; three SNPs, rs329068902 (+556C > A), rs318859497 (+573G > A), and rs337682650 (+574T > G), were identified in the first intron, and rs342747498 (+2096T > G) was identified in the fifth exon (Figure 2a). Significant differences (*p* < 0.05) in allele frequency were found between large white and Mi pigs in six of the seven SNPs (Table 1). The number of each SNP genotype was provided in Supplementary Table S5. In Large White pigs, genotypic distributions of the seven SNPs were in accordance with the Hardy–Weinberg equilibrium (HW *p* > 0.05) (Table 2). In Mi pigs, only rs342747498 was in accordance with the Hardy–Weinberg equilibrium (HW *p* = 1), while the other six SNPs deviated from the Hardy–Weinberg equilibrium (HW *p* < 0.05) (Table 3). Linkage disequilibrium analyses showed that six SNPs, except for rs318859497, were completely linked in Large White pigs (Figure 2b); while six SNPs, except for rs342747498, were completely linked in Mi pigs (Figure 2c).

### 3.4. Promoter Identification of Porcine HSD17B14 Gene

**Figure 3:**
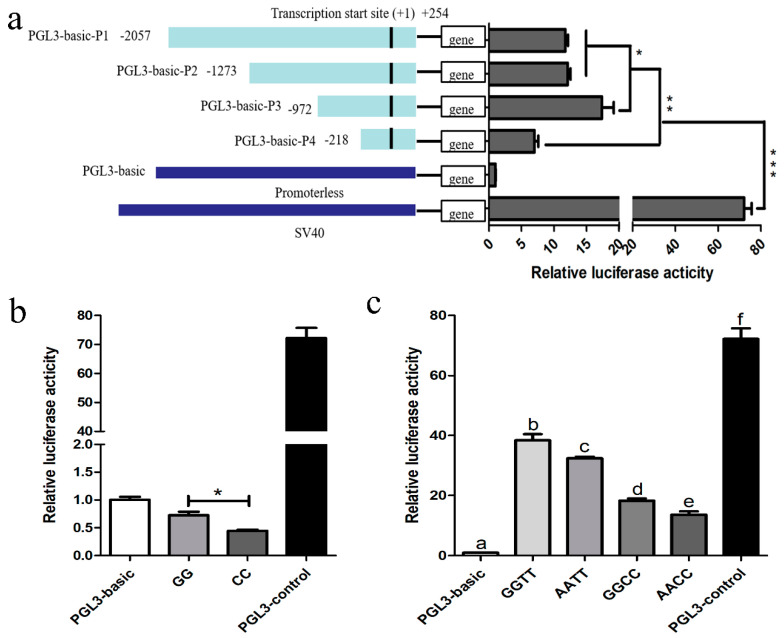
Luciferase assays for porcine *HSD17B14* promoter activity analyses. The resulting firefly luciferase activity was normalized to Renilla luciferase activity and the relative values are presented as fold induction over the activity of the pGL3-basic vector. The basic activity value of positive control PGL3-basic was set as 1. PGL3-basic as a negative control and PGL3-control as a positive control. The relative luciferase activity values represent the mean ± SEM of three independent experiments. (**a**) Four luciferase reporter plasmids expressing successive truncations of the *HSD17B14* promoter sequence were constructed and transfected into 293T cells. (**b**) Luciferase reporter gene assays of porcine *HSD17B14* alleles containing rs342163057 (−850G > C). Two genotype luciferase reporter vectors of the *HSD17B14* −949 to −701 bp sequence were constructed and transfected into 293T cells. (**c**) Luciferase reporter gene assays of porcine *HSD17B14* alleles containing rs329427898 (−683A > G) and rs319864566 (−502T > C). Four genotype luciferase reporter vectors of the *HSD17B14* −720 to −394 bp sequence were constructed and transfected into 293T cells. The relative luciferase activity values represent the mean ± SEM of three independent experiments. Statistical differences in luciferase activity were assessed using the one-way ANOVA analysis, * *p* < 0.05, ** *p* < 0.01, *** *p* < 0.001. Different letters (a, b and c) indicate that the difference is significant (*p* < 0.05).

The proximal minimal promoter of the porcine *HSD17B14* gene was isolated to determine the SNPs that are responsible for influencing the function of the promoter. The luciferase reporter plasmids, named PGL3-basic-P1 (−2057/+254), PGL3-basic-P2 (−1273/+254), PGL3-basic-P3 (−972/+254), PGL3-basic-P4 (−218/+254), were transfected into 293T cells. The luciferase activity of plasmids with one of four *HSD17B14* promoter fragments was greater than that of the pGL3-basic group (*p* < 0.01) and less than that of the pGL3-control group (*p* < 0.01). The luciferase activity of PGL3-basic-P3 was greater than that of PGL3-basic-P1 (*p* = 0.0402) or PGL3-basic-P2 (*p* = 0.0496) (Figure 3). Moreover, the luciferase activity of PGL3-basic-P1, PGL3-basic-P2, or PGL3-basic-P3 was greater than that of PGL3-basic-P4 (*p* < 0.05). These results show that the core promoter region of the *HSD17B14* gene is located between −972 bp and −218 bp, whereas a negative regulatory promoter region is located between −1273 bp and −972 bp.

### 3.5. Promoter Activity Analyses of Porcine HSD17B14 Gene

In the seven SNPs of the *HSD17B14* gene, rs342163057 (−850G > C) and rs329427898 (−683A > G) are located at downstream of the promoter region and rs319864566 (−502T > C) is located at the core promoter region. Promoter activity of different genotypes of rs342163057 (−850G > C), rs329427898 (−683A > G), and rs319864566 (−502T > C) in the porcine *HSD17B14* gene were analyzed using a dual-luciferase reporter assay system. As shown in Figure 3b, the luciferase activity was greater in plasmids of genotype GG than that of plasmids of genotype CC of rs342163057 (*p* < 0.05). In Figure 3c, the luciferase activities of plasmids with combined genotype GGTT, AATT, GGCC, or AACC of rs329427898 (−683A > G) and rs319864566 (−502T > C) were greater than those of the negative control pGL3-basic group (*p* < 0.01) but less than those of the pGL3-control group (*p* < 0.01). Luciferase reporter plasmids with combined genotype AATT had less (*p* = 0.0451) luciferase activity than that of plasmids with combined genotype GGTT. In addition, the plasmids with combined genotype AACC had lower (*p* = 0.026) luciferase activities than those of plasmids with combined genotype GGCC. These results suggest that the plasmids with genotype AA had lower luciferase activities than those of plasmids with genotype GG of rs329427898 (−683A > G). At the same time, the plasmids with combined genotype GGCC have lower (*p* = 0.0007) luciferase activities than those of plasmids with GGTT, while the plasmids with combined genotype AACC had lower (*p* = 0.0001) luciferase activity than that of plasmids with combined genotype AATT of rs329427898 (−683A > G) and rs319864566 (−502T > C). These results suggest that the plasmids with genotype CC had lower luciferase activities than those of plasmids with genotype TT of rs319864566 (−502T > C).

### 3.6. Differential Expression and Function of Phe73Val of HSD17B14 in Porcine Ovarian Granulosa Cells

**Figure 4:**
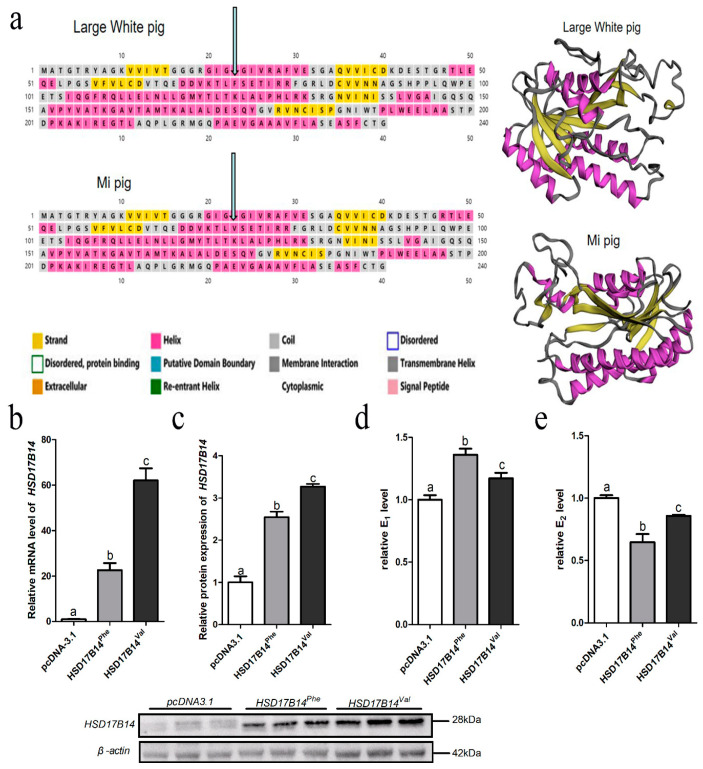
Differential expression and function of the Phe73Val of porcine *HSD17B14* gene in porcine ovarian granulosa cells (GCs). Bioinformatics website PSIpred-MEMSAT3 was used to analyze the structural differences of proteins and build a three-dimensional model. (**a**) Secondary structure of protein of in Large White pigs and three dimensional image construction based on the secondary structure of protein of Large White pigs. Secondary structure of protein in Mi pigs and three dimensional image construction based on the secondary structure of protein of Mi pigs. (**b**) RT-qPCR analyses of *HSD17B14* mRNA expression in porcine GCs transfected with *HSD17B14*^Phe^, *HSD17B14*^Val^ or pcDNA3.1(*+*). The mRNA levels were normalized to *GAPDH*. (**c**) Western blot analyses of *HSD17B14* protein expression in porcine GCs transfected with *HSD17B14*^Phe^, *HSD17B14*^Val^ and pcDNA3.1(*+*). The protein levels were normalized to β-actin. (**d**) E_1_ concentration (IU/L). The cell supernatants were collected 48 h after transfection and examined using porcine E_1_ ELISA kits. (**e**) E_2_ concentration (IU/L). The cell supernatants were collected 48 h after transfection and examined using porcine E_2_ ELISA kits. Each experiment was repeated three times and data are shown as means ± S.E.M. *p*-values were calculated by *t*-tests, different letters (a, b and c) indicate that the difference is significant (*p* < 0.05).

The SNP rs342747498 (T/G) is located in exon five of the porcine *HSD17B14* gene, according to the information in Ensembl (https://www.ensembl.org/index.html). It causes a mutation from phenylalanine (F) to valine (V) at the seventy-third amino acid of the *HSD17B14* protein. Since the genotype of rs342747498 was TT in Large White pigs and GG in Mi pigs, the protein sequences of *HSD17B14* were totally different between the Large White (*HSD17B14*^Phe^) and Mi (*HSD17B14*^Val^) pigs. Predicted protein structure figures show that the three-dimensional structures of the *HSD17B14* protein are different between Large White and Mi pigs (Figure 4a,b). The change of the seventy-third amino acid increases strand and decreases helix structure (Figure 4a,b).

Vectors pcDNA3.1 (empty vector), *HSD17B14*^Phe^ or *HSD17B14*^Val^ were transfected into porcine GCs. Subsequently, the mRNA expression level of *HSD17B14*^Val^ was significantly greater than that of *HSD17B14*^Phe^ (*p* < 0.05), whereas the mRNA expression level of *HSD17B14*^Val^ or *HSD17B14*^Phe^ was greater *p* < 0.05) than that of pcDNA3.1 (Figure 4c). The assays of the Western blot showed that the protein expression level of *HSD17B14*^Val^ was significantly greater than that of *HSD17B14*^Phe^ (*p* < 0.05), while the protein expression levels of both *HSD17B14*^Val^ and *HSD17B14*^Phe^ were greater (*p* < 0.05) than those of pcDNA3.1 (Figure 4d,e). In addition, estradiol concentrations in GCs in the *HSD17B14*^Val^ group were significantly greater (*p* < 0.05) than those of GCs in the *HSD17B14*^Phe^ group (Figure 4d), while estrone concentrations in GCs in the *HSD17B14*^Val^ group were significantly less (*p* < 0.05) than those of GCs in the *HSD17B14*^Phe^ group (Figure 4e).

### 3.7. Effect of Phe72Val of HSD17B14 Gene on Apoptosis of Porcine Ovarian Granulosa Cells

**Figure 5:**
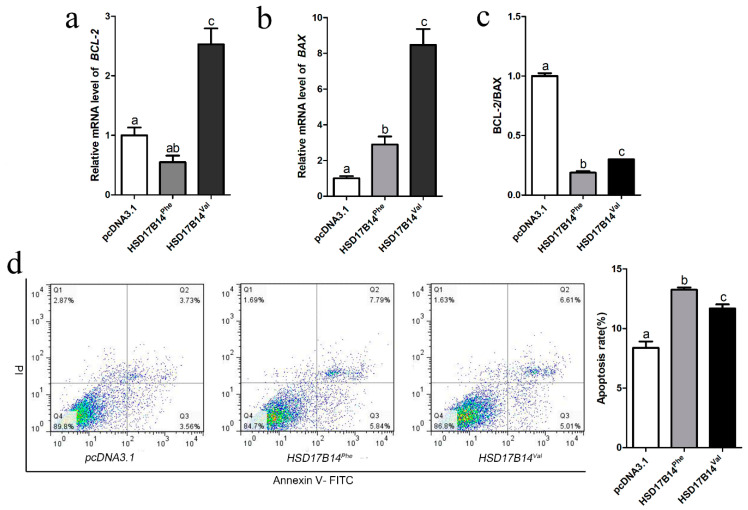
Effect of overexpression of the porcine *HSD17B14* gene Phe73Val on apoptosis of ovarian granulosa cells (GCs). (**a**,**b**) RT-qPCR analyses of *BCL-2* and *BAX* mRNA expression in porcine GCs transfected with *HSD17B14*^Phe^, *HSD17B14*^Val^ or pcDNA3.1(*+*). The mRNA levels were normalized to *GAPDH*. (**c**) *BCL-2/BAX* ratio. (**d**) Fluorescence-activated cell sorting (FACS) analyses of the apoptosis rates of porcine GCs when *HSD17B14*^Val^ and *HSD17B14*^Phe^ were overexpressed. Each experiment was repeated three times and data are shown as means ± S.E.M. *p*-values were calculated by *t*-test, different letters (a, b and c) indicate that the difference is significant (*p* < 0.05).

The metabolism of estradiol is closely related to the apoptosis of porcine ovarian GCs [34]. To investigate whether there is a different effect on the apoptosis of porcine GCs between the *HSD17B14*^Val^ and *HSD17B14*^Phe^ proteins, a *HSD17B14*^Phe^ or *HSD17B14*^Val^ overexpression vector was transfected into porcine GCs. RT-qPCR of *BAX* and *BCL-2* genes showed that the ratio of *BCL-2/BAX* was significantly less (*p* < 0.05) in the *HSD17B14*^Phe^ group than in the *HSD17B14*^Val^ group, while it was less (*p* < 0.05) in the *HSD17B14*^Phe^ or *HSD17B14*^Val^ group than in the pcDNA3.1 group (Figure 5c). The result showed that both *HSD17B14*^Phe^ and *HSD17B14*^Val^ caused apoptosis of GCs, but the apoptosis level caused by *HSD17B14*^Val^ was significantly less (*p* < 0.05) than that caused by *HSD17B14*^Phe^. A fluorescence activated cell sorting (FACS) analysis also revealed that the GCs in the *HSD17B14*^Phe^ group had a greater (*p* < 0.05) apoptosis rate than the GCs in the *HSD17B14*^Val^ group (Figure 5d).

## 4. Discussion

### 4.1. Tissue Expression Profile of HSD17B14 Gene in Gilts

Gene expression analyses and tissue-specific expression profiling might be useful for functional research and gene mapping. [35,36]. Estrus behaviors in gilts are complex traits associated with estrogen receptors [4]. The *HSD17B14* gene was found to be widely expressed in different tissues of gilts in the present study. However, this gene was differently expressed between the diestrus and estrus periods in the ovaries, kidneys, and brains of gilts, which suggests that *HSD17B14* gene may have an important role in regulating the expression of estrus in various tissues. Moreover, the mRNA expression level of the *HSD17B14* gene was greater in the diestrus period than in the estrous period. The Western blot analyses also revealed that the expression level of the *HSD17B14* protein was greater in Mi gilts than in Large White gilts. The *HSD17B14* protein converts 17β-OH steroids, for example estradiol (E_2_), into estrone (E_1_) both in vivo and in vitro [14]. It plays a key role in estradiol metabolism during the diestrus period. Previous studies have found that the *HSD17B14* gene was differentially expressed during the diestrous and estrus periods between Large White and Mi gilts [24]. Thus, the porcine *HSD17B14* gene could affect the estrus of gilts by regulating the transformation of estradiol to estrone.

### 4.2. Prediction and Identification of the Core Promoter in the Porcine HSD17B14 Gene

As an important component of a gene, the promoter is a cis-acting element for the regulation of gene expression in eukaryotes, controlling the initiation location and abundance of gene expression [37]. A CpG island could influence the chromatin structure and regulate gene activity [38]. In the present study, the promoter region of the porcine *HSD17B14* gene was identified and located at a CpG island (−667 bp and −562 bp) (Supplementary Table S4), which is able to regulate the expression of the *HSD17B14* gene by methylation. By analyzing the results of luciferase assays, the porcine *HSD17B14* gene not only had a positive regulatory promoter region between −972 bp and −218 bp, but also a negative regulatory promoter region between −1273 bp and −972 bp. These results suggest that these two promoter regions could play a role in the regulation of gene expression [39]. Moreover, these promoter regions could serve as regulatory elements for the assembly of transcription machinery, especially through combination with RNA polymerases [40], for promoting accurate initiation of transcription. Therefore, prediction and identification of the core promoter is a significant step in unravelling the mechanisms of the *HSD17B14* gene transcriptional and expressional regulation.

### 4.3. Screening of SNPs in Porcine HSD17B14 Gene Associated with Estrus Behavior in Gilts

Our previous studies found that Chinese Mi gilts had more intense estrus behaviors and greater estradiol-17β concentrations than Large White gilts [23]. The expression level of the porcine *HSD17B14* gene was significantly different between the two stages of the estrus cycle and between the two pig breeds [24]. In the present study, a total of seven linked SNPs were found in the promoter, exon, and 3’UTR regions of the porcine *HSD17B14* gene. Three SNPs in the promoter region caused the different expression level of the porcine *HSD17B14* gene between Mi and Large White gilts. Two SNPs in the first intron acted on the transcription of the *HSD17B14* gene through linkage with the SNPs in the promoter region in Large White gilts. However, rs318859497 was also linked with the SNPs in the promoter region, in addition to rs329068902 and rs337682650 in Mi pigs. In addition, as the result of a missense mutation, an encoded amino acid changed from phenylalanine (F) to valine (V) in the fifth exon. Previous studies had shown that certain specific missense mutations not only affected the function of the target protein and the incidence of disease [41], but also caused changes in the structure and function of the protein [42]. For example, low stability of the mRNA structure may reduce the shear efficiency or stability, thereby reducing protein expression or enzyme activity [43].

### 4.4. Promoter Activity Analyses of Porcine HSD17B14 Gene

Polymorphisms in the 5’-UTR of a gene could affect its transcription [44]. In the present study, we found that three SNPs in the 5’-UTR of the *HSD17B14* gene were not located in the CpG island. Because the luciferase activities of plasmids with GG and CC genotypes were less than those of the negative control—the pGL3-basic group (*p* < 0.05)—there was a transcription-inhibiting element that binds to CC and GG genotypes (the binding energy of the CC genotype was greater than that of GG), resulting in the luciferase activity of rs342163057 (−850G > C) being less than that of PGL3-basic. For the other two SNPs rs329427898 (−683A > G) and rs319864566 (−502T > C), the combined genotypes (GGTT, AATT, GGCC, and AACC) showed a progressive increase and significant differences between them. The luciferase activities of plasmids with GG and TT genotypes were greater than those of plasmids with AA and CC genotypes, which suggests that these two SNPs can affect the promoter activity of the *HSD17B14* gene. In addition, rs342163057 (−850G > C), rs329427898 (−683A > G) and rs319864566 (−502T > C) were located in the positive regulatory region in the promoter. Compared to rs329427898 (−683A > G), rs319864566 (−502T > C) is the dominant SNP for regulating the porcine *HSD17B14* gene.

In the present study, we predicted several transcription factors, such as STAT4, TFII-1, c-Ets-1, and NF-AT2 (Supplementary Table S3) for the porcine *HSD17B14* gene. They were reported to have important influences in tumorigenesis and cell growth [45].

### 4.5. Differential Expression and Function of Phe73Val of HSD17B14 in Porcine Ovarian Granulosa Cells

A missense mutation is an inheritable alteration in the sequence of the genetic material of an organism [46]. In the human *HSD17B14* gene, the catalytic activity of the enzyme had a larger change than before the mutation [47]. In the present study, we found a missense mutation in the porcine *HSD17B14* gene in the fifth exon in Large White and Mi gilts. The missense mutation could lead to changes in the structural dynamics, which are determinant of the functional significance of missense variants [42]. Protein three-dimensional structure prediction showed that the missense mutation brought obvious changes in each region of the *HSD17B14* protein, including strand and helix structure. These changes could affect the enzyme activity of the *HSD17B14* protein [46], which might be the cause of the difference of estrus behavior between Chinese Mi and European Large White gilts.

In addition, both mRNA and protein levels of *HSD17B14* were significantly lower in the overexpression vector *HSD17B14*^Phe^ group than in the *HSD17B14*^Val^ group in the present study. These results indicate that more highly expressed *HSD17B14*^Val^ did not degrade more estradiol into estrone, while the relatively lesser expressed *HSD17B14*^Phe^ degraded more estradiol into estrone, suggesting the protein activity of *HSD17B14*^Phe^ was greater than that of *HSD17B14*^Val^. In this situation, the low protein activity of *HSD17B14*^Val^ did not cause a significant reduction in estradiol in porcine ovarian GCs, and the feedback regulation would increase the mRNA and protein levels of *HSD17B14*^Val^. These results further illustrate why the expression level was greater for the *HSD17B14* gene in Mi gilts than in Large White gilts. The results also explained why Chinese Mi gilts had better estrus behavior traits than Large White gilts.

### 4.6. The Effect of HSD17B14 Phe73Val for Apoptosis of Ovarian Granulosa Cells by Degraded E_2_

Estrogen is necessary for the proliferation and apoptosis of porcine GCs [48,49]. In a previous study, E_2_ promoted the proliferation of porcine GCs in a time- and dose-dependent way [48]. Similarly, E_2_ abolished gonadotropin-mediated pro-apoptotic signals in human primary granulosa lutein cells [50]. In addition, E_2_ also prevented H_2_O_2_-induced apoptosis of large luteal and granulosa cells of pigs [51]. In the present study, porcine *HSD17B14*^Phe^ degraded more estrogen than *HSD17B14*^Val^. Thus, apoptosis of pig ovarian granulocytes was greater in *HSD17B14*^Phe^ than in *HSD17B14*^Val^. Follicle development is closely associated with the development of GCs [52]. Therefore, *HSD17B14*^Phe^ could have a negative impact on ovarian development and the reproductive performance of female pigs, which could inhibit estrus signs of gilts.

**Figure 6:**
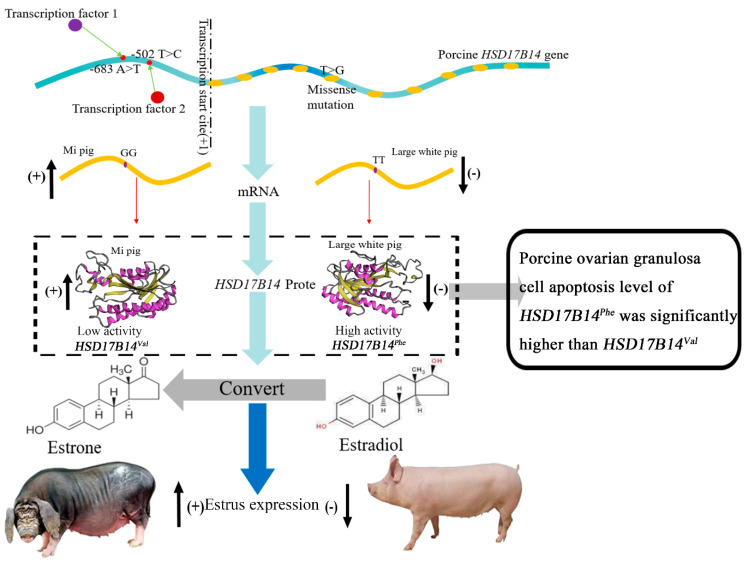
Regulation mechanism of the porcine *HSD17B14* gene on estrus signs in Large White and Mi gilts.

Our previous study found that the expression level of the *HSD17B14* gene in the follicles was greater in Mi gilts than in Large White gilts during diestrous, but was lower in Mi gilts than in Large White gilts during estrus [24]. The porcine *HSD17B14* protein was mainly involved in the transformation from high-efficient E_2_ to low-efficient E_1_ in the estrogen metabolism pathway [14]. Previous studies demonstrated that the concentrations of estradiol were positively associated with estrus signs of gilts [2,9]. Therefore, the rs329427898 and rs319864566 genotypes in the *HSD17B14* gene could affect the binding of RNA polymerase and transcription factors to the promoter region, influencing its expression and the ability to degrade estradiol to estrone. In addition, although Mi gilts showed an increase in protein and mRNA expression levels, the missense mutation of Phe73Val in the fifth exon weakens the ability of the protein to degrade estradiol to estrone, which would make the estrus signs more obvious and longer lasting in Mi gilts (Figure 6). Furthermore, this missense mutation in Mi gilts decreases the apoptosis level of ovarian GCs compared to Large White gilts, which has a profound impact on the estrus sign traits of the two pig breeds. These might be the cause of the difference of estrus signs between the Chinese Mi and European Large White gilts. Importantly, the results in the present study provide a molecular marker (*HSD17B14*^Val^) that can be used to screen gilts with better estrus sign traits. Better estrus traits could also improve the accuracy of estrus detection, which increases the efficiency of pig reproduction.

## 5. Conclusions

The promoter activity analyses suggest that the core promoter region of the porcine *HSD17B14* gene is located between −972 bp and −218 bp from the initiation site of transcription. A negative regulatory promoter region was found between −1273 bp and −972 bp. The luciferase reporter plasmids with the GG genotype of rs329427898 had greater promoter activities than plasmids with the AA genotype; plasmids with the TT genotype of rs319864566 had greater promoter activities than plasmids with the CC genotype, which is consistent with the estrus difference between Large White and Mi gilts in vivo. In addition, rs342747498 located in exon five of the *HSD17B14* gene caused a missense mutation, which lead to phenylalanine (F) being changed to valine (V) at seventy-third amino acid position in the *HSD17B14* protein. This had a profound effect on the mRNA and protein expression levels of the porcine *HSD17B14*. These results indicate that these SNPs in the *HSD17B14* gene could be candidate molecular markers for estrus signs in pigs.

## Figures and Tables

**Table 1:** The allele frequencies of single nucleotide polymorphisms (SNPs) in the porcine *HSD17B14* gene for Large White and Mi pigs.

SNPs	Region	SNP Site	Allele	Large White Pigs/Mi Pigs	ꭓ^2^	*p*-Value
rs342163057	5’UTR	G > C	G	0.477/0.720	5.369	0.020 *
			C	0.523/0.280		
rs329427898	5’UTR	A > G	A	0.363/0.767	14.415	0.000 ***
			G	0.637/0.233		
rs319864566	5’UTR	T > C	T	0.400/0.090	12.141	0.000 ***
			C	0.600/0.910		
rs329068902	Intron 1	C > A	C	0.700/0.750	0.198	0.656
			A	0.300/0.250		
rs318859497	Intron 1	C > T	C	1.000/0.750	14.326	0.000 ***
			T	0.000/0.250		
rs337682650	Intron 1	G > A	G	0.820/0.170	42.688	0.000 ***
			A	0.180/0.830		
rs342747498	Exon 5	T > G	T	0.400/0.078	14.411	0.000 ***
			G	0.600/0.922		

Χ^2^: chi-square value; * *p* < 0.05, *** *p* < 0.001.

**Table 2:** The HW and MAF of SNPs in porcine *HSD17B14* gene in Large White pigs.

SNPs	Mutation Position	ObsHET	PredHET	HW P	%Geno	MAF
rs342163057	6:54145324	0.54	0.484	0.6479	100	0.41
rs329427898	6:54145156	0.54	0.484	0.6479	100	0.41
rs319864566	6:54144975	0.54	0.000	1.0000	100	0.41
rs329068902	6:54143919	0.54	0.476	0.5589	100	0.39
rs318859497	6:54143903	0.00	0.000	1.0000	100	0.00
rs337682650	6:54143902	0.54	0.476	0.5589	100	0.39
rs342747498	6:54142047	0.54	0.476	0.5589	100	0.39

MAF: minor allele frequency; HW P: *p*-value of Hardy-Weinberg balance.

**Table 3:** The HW and MAF of SNPs in porcine *HSD17B14* gene in Mi pigs.

SNPs	Mutation Position	ObsHET	PredHET	HW P	%Geno	MAF
rs342163057	6:54145324	0.714	0.493	0.0043	100	0.439
rs329427898	6:54145156	0.714	0.493	0.0043	100	0.439
rs319864566	6:54144975	0.714	0.493	0.0043	100	0.439
rs329068902	6:54143919	0.714	0.493	0.0043	100	0.439
rs318859497	6:54143903	0.755	0.499	9 × 10^−4^	100	0.480
rs337682650	6:54143902	0.714	0.493	0.0043	100	0.439
rs342747498	6:54142047	0.020	0.020	1.0000	100	0.010

MAF: minor allele frequency; HW P: *p*-value of Hardy-Weinberg balance.

## Supplementary Materials

The following are available online at https://www.mdpi.com/2218-273X/10/11/1545/s1, Table S1: Primer information of SNP identification for porcine HSD17B14 gene, Table S2: Primer information of plasmid construction and RT-qPCR for porcine HSD17B14 gene, Table S3: Change of transcription factor-binding sites before and after the SNPs rs329427898 and rs319864533 mutation in the promoter region of the porcine HSD17B14 gene, Table S4: The prediction of CpG island in the promoter of porcine HSD17B14 gene by Meth-Primer 2.0, Table S5: Allelic distribution of SNPs of porcine HSD17B14 gene in the Large White and Mi pigs, Table S6: The data of estrus expression in Large White and Mi pigs.
