# Supplementary material for: Identification of Functional Single Nucleotide Polymorphisms in Porcine HSD17B14 Gene Associated with Estrus Behavior Difference between Large White and Mi Gilts
Source: Biomolecules. 2020 Nov 12;10(11):1545. doi: 10.3390/biom10111545 (PMC7697482; doi:10.3390/biom10111545)
Supplement: Supplementary file 1 [file biomolecules-10-01545-s001.zip › Supplementary-20201109.docx]

**Identification of functional single nucleotide polymorphisms in porcine *HSD17B14* gene associated with estrus behavior difference between Large White and Mi gilts**

**Siyuan Gao^1^, Ruixin Tao^1^, Xian Tong^1^, Qinglei Xun^1^, Zhao Jing^1^, Yanli Guo^1^, Allan P. Schinckel^2^, Bo Zhou^1,^ ***

^1^College of Animal Science and Technology, Nanjing Agricultural University, Nanjing 210095, China

^2^Department of Animal Sciences, Purdue University, West Lafayette, IN 47907-2054, USA

*Corresponding author: email: [zhoubo@njau.edu.cn](mailto:zhoubo@njau.edu.cn)

**Supplementary:**

Table S1. Primer information of SNP identiﬁcation for porcine *HSD17B14* gene.

| Primer | Primer sequence (5'-3') | | Product Size (bp) | | | Start | Stop | | Usage |
| --- | --- | --- | --- | --- | --- | --- | --- | --- | --- |
| HSD17B14-1 | | F:TTCACACGGTTACCAGGGGG | | 835 | -2051 | | | -2032 | PCR |
|  |  | R:CACTGAGCAAGGGCAGGGAC | |  | -1236 | | | -1217 |  |
| HSD17B14-2 | | F:GTCTGTTAGAAGTCGAAGGTA | | 950 | -1371 | | | -1352 | PCR |
|  |  | R:CTCGGACTAGAAAAAAGAAG | |  | -436 | | | -427 |  |
| HSD17B14-3 | | F:GTTCCACCGTTGCTGTCAAA | | 880 | -648 | | | -649 | PCR |
|  |  | R:AGAGGCCACGCCTCCTCGTC | |  | 213 | | | 232 |  |
| HSD17B14-4 | | F:CGCCCCCAAGATCTCCTCCA | | 912 | 79 | | | 98 | PCR |
|  |  | R:CCCCCGTCACGATTACCACC | |  | 971 | | | 990 |  |
| HSD17B14-5 | | F:AGCCCCTATTTATGGTTCGA | | 271 | 837 | | | 856 | PCR |
|  |  | R:CTTCGTCCCACACCCATTAG | |  | 1091 | | | 1100 |  |
| HSD17B14-6 | | F:CCACAATGTCCTGTCCCCTC | | 272 | 2312 | | | 2331 | PCR |
|  |  | R:AGAAATGTAGAAACTGAGGC | |  | 2564 | | | 2583 |  |
| HSD17B14-7 | | F:ACCCTCTCGTCTGCCACTCC | | 461 | 7590 | | | 7609 | PCR |
|  |  | R:TGAACCCGCACCTCCACCGT | |  | 8031 | | | 8050 |  |
| HSD17B14-8 | | F:AAGGGCTGAGACCGAAGGCA | | 655 | 17998 | | | 18017 | PCR |
|  |  | R:CCCAGTCTTGAAGGCTCC | |  | 18635 | | | 18652 |  |
| HSD17B14-9 | | F:CCAAAATCCGAGAGGGCACG | | 445 | 18776 | | | 18795 | PCR |
|  |  | R:TGGGGTGGTCTGGACATTC | |  | 19202 | | | 19220 |  |
| HSD17B14-10 | | F:GCAAACTCAGTCTCCCAAAT | | 339 | 18336 | | | 18355 | PCR |
|  |  | R:CACAGCAATGCGGAATCTAA | |  | 18655 | | | 18674 |  |

**Table S2. Primer information of** **plasmid construction and RT-qPCR for porcine *HSD17B14* gene.**

| Primer | Primer sequence (5'-3') | | | Product Size (bp) | | | | | | | Start | Stop | | | | Usage | | | |
| --- | --- | --- | --- | --- | --- | --- | --- | --- | --- | --- | --- | --- | --- | --- | --- | --- | --- | --- | --- |
| HSD17B14-P1 | | | F:CGAGCTCGATTTTATTCACACGGTTACC | | | 2311 | | -2057 | | | | | | -2038 | | Promoter vector | | | |
|  |  |  | R:CCCAAGCTTGGACTGTAGCAGCGTGACTC | | | |  | | 235 | | | | | | 254 | | construction | | |
| HSD17B14-P2 | | | F:CGAGCTCGGTCCTGTCTGTTAGAAGTCG | | | | 1572 | | -1273 | | | | | | -1254 | | Promoter vector | | |
|  |  |  | R:CCCAAGCTTGGACTGTAGCAGCGTGACTC | | | |  |  | 235 | | | | | | 254 | | construction | | |
| HSD17B14-P3 | | | F:CGAGCTCGGCAATTTTCTCTCTTCGCTA | | | | 808 | | -972 | | | | | | -953 | | Promoter vector | | |
|  |  |  | R:CCCAAGCTTGGACTGTAGCAGCGTGACTC | | | |  |  | 235 | | | | | | 254 | | construction | | |
| HSD17B14-P4 | | | F:CGAGCTCGCCGACGACCGCCAGAAACCC | | | | 472 | | -218 | | | | | | -199 | | Promoter vector | | |
|  |  |  | R:CCCAAGCTTGGACTGTAGCAGCGTGACTC | | | |  |  | 235 | | | | | | 254 | | construction | | |
| HSD17B14-P5 | | | F:CGAGCTCGTCCTTCACTTCATCCTCAAA | | | | 249 | | -949 | | | | | | -930 | | SNP vector | | |
|  |  |  | R:CCCAAGCTTGTTCTGCCATCCATACCCTG | | | |  |  | -720 | | | | | | -701 | | construction | | |
| HSD17B14-P6 | | | F:CGAGCTCGCAGGGTATGGATGGCAGAAC | | | | 327 | | -720 | | | | | | -701 | | SNP vector | | |
|  |  |  | R:CCCAAGCTTTGGTAAATAAGGGGTCGTGT | | | |  |  | -414 | | | | | | -395 | | construction | | |
| HSD17B14 | | | F: TGGACCCCGCTGTGGGAAGA | | | |  | |  | | | | | |  | | RT-qPCR | | |
|  |  |  | R: GCGTGCCCTCTCGGATTTTG | | | |  |  |  | | | | | |  | |  |  |  |
| GAPDH | | | F: GATGGTGAAGGTCGGAGTG | | | |  | |  | | | | | |  | | RT-qPCR | | |
|  |  |  | R: CGAAGTTGTCATGGATGACC | | | |  | |  | | | | | |  | |  |  |  |
| BCL-2 | | | F: TTCTTTGAGTTCGGTGGGG | | | |  | |  | | | | | |  | | RT-qPCR | |  |
|  |  |  | R: CCAGGAGAAATCAAATAGAGGC | | | |  | |  | | | | | |  | |  |  |  |
| BAX | | | F: CCGAAATGTTTGCTGACG | | | |  | |  | | | | | |  | | RT-qPCR | | |
|  |  |  | R: AGCCGATCTCGAAGGAAGT | | | |  | |  | | | | | |  | |  |  |  |
| HSD17B14Phe  HSD17B14Val | | F: CCCAAGCTTCTTTCTGTCTGTTTGGAGTT | | |  | | | | |  | | |  | | | | | Vector construction | |
| HSD17B14Va | | R:GCTCTAGAGCATTTGGGAGACTGAGTTTGC | | |  | | | | |  | | |  | | | | |  |  |

**Table S3. Change of transcription factor-binding sites before and after the SNPs rs329427898 and rs319864533 mutation in the promoter region of the porcine *HSD17B14* gene.**

| SNP | Haplotypes and sequence | | Transcription factor | | Predicted site sequence | | Dissimilarity | RE equally | | RE query | |
| --- | --- | --- | --- | --- | --- | --- | --- | --- | --- | --- | --- |
| rs329427898 | | **A**:CGCTTGA**A**TTCCTGGG | | GR-beta | | GAATTCCT | 1.68% | | 0.06055 | | 0.06098 |
|  |  |  |  | TFII-I | | AATTCC | 9.51% | | 0.11353 | | 0.119 |
|  |  |  |  | STAT4 | | AATTCC | 1.47% | | 0.03027 | | 0.0313 |
|  |  |  |  | c-Ets-1 | | ATTCCTG | 4.15% | | 0.00378 | | 0.00346 |
|  |  | **G**:CGCTTGA**G**TTCCTGGG | | TFII-I | | AGTTCC | 14.27% | | 0.09082 | | 0.08852 |
|  |  |  |  | STAT4 | | AGTTCC | 1.47% | | 0.03027 | | 0.02724 |
|  |  |  |  | c-Ets-1 | | GTTCCTG | 3.46% | | 0.00946 | | 0.00809 |
| rs319864566 | | **T**:CGCTTGA**T**TTCCTGGG | | NF-AT2 | | CTTGATTTCC | 13.96% | | 0.00078 | | 0.00129 |
|  |  |  |  | TFII-I | | ATTTCC | 4.76% | | 0.02344 | | 0.01021 |
|  |  |  |  | STAT4 | | ATTTCC | 0.00% | | 0.00391 | | 0.00172 |
|  |  |  |  | c-Ets-1 | | TTTCCTG | 1.38% | | 0.00293 | | 0.00928 |
|  |  |  |  | STAT1beta | | ATTTCCTGGG | 5.80% | | 0.00124 | | 0.00076 |
|  |  |  |  | IRF-1 | | TTTCCTGGG | 7.48% | | 0.00116 | | 0.00184 |
|  |  |  |  | GR-beta | | TGATT | 4.20% | | 0.0625 | | 0.03159 |
|  |  |  |  | NF-AT1 | | TTGATTTCC | 9.04% | | 0.00183 | | 0.0027 |
|  |  | C:CGCTTGA**C**TTCCTGGG | | TFII-I | | ACTTCC | 14.27% | | 0.04688 | | 0.0236 |
|  |  |  |  | STAT4 | | ACTTCC | 2.94% | | 0.02344 | | 0.02789 |
|  |  |  |  | c-Ets-1 | | CTTCCTG | 0.00% | | 0.00195 | | 0.00469 |
|  |  |  |  | Elk-1 | | CTTCCTGGG | 2.30% | | 0.00073 | | 0.00201 |
|  |  |  |  | AP-1 | | TGACTTCCT | 13.47% | | 0.00195 | | 0.00095 |
|  |  |  |  | c-Jun | | TGACTTC | 5.19% | | 0.00488 | | 0.00227 |

Random Expectation (RE) gives the number of expected occurrences of the match in a random sequence of the same length as the query sequence according to the dissimilarity index. Two models are considered:

RE equally: Equiprobability for the 4 nucleotides; RE query: Estimate the nucleotide probability as the nucleotide frequencies in the query sequence

**Table S4. The prediction of CpG island in the promoter of porcine *HSD17B14* gene by Meth-Primer 2.0**

| CpG island | Size(bp) | | | Start | End | |
| --- | --- | --- | --- | --- | --- | --- |
| Island 1 | | 212 | -1204 | | | -1084 |
| Island 2 | | 106 | -667 | | | -562 |
| Island 3 | | 238 | -40 | | | 198 |

**Table S4. Allelic distribution of SNPs of porcine *HSD17B14* gene in the Large White and Mi pigs.**

| SNPs | Breed | Genotype* |
| --- | --- | --- |

| rs342163057 | Large White pigs | GG(16) | GC(27) | CC(7) |
| --- | --- | --- | --- | --- |
|  | Mi pig | GG(3) | GC(37) | CC(10) |
| rs329427898 | Large White pigs | GG(7) | AG(27) | AA(16) |
|  | Mi pig | GG(10) | AG(34) | AA(4) |
| rs319864566 | Large White pigs | CC(7) | CT(27) | TT(16) |
|  | Mi pig | CC(10) | CT(36) | TT(4) |
| rs329068902 | Large White pigs | CC(17) | CA(27) | AA(6) |
|  | Mi pig | CC(4) | CA(36) | AA(10) |
| rs318859497 | Large White pigs | CC(50) | CT(0) | TT(0) |
|  | Mi pig | CC(5) | CT(38) | TT(7) |
| rs337682650 | Large White pigs | AA(6) | AG(27) | GG(17) |
|  | Mi pig | AA(10) | AG(36) | GG(4) |
| rs342747498 | Large White pigs | TT(17) | TG(37) | GG(6) |
|  | Mi pig | TT(0) | TG(1) | GG(49) |

*: Genotype and the number of pigs with the genotype.

**Table S5. The data of estrus expression in Large White and Mi pigs.**

| Item | breed | | |
| --- | --- | --- | --- |
|  | Large white pigs | Mi pigs | P value |
| Color of the vulva | 1.03±0.06 | 1.77±0.07 | 0.000 |
| Color of vaginal mucus | 1.47±0.06 | 1.93±0.07 | 0.000 |
| Amount of mucus | 1.41±0.08 | 1.45±0.09 | 0.745 |
| Viscosity of mucus | 1.41±0.08 | 1.70±0.09 | 0.019 |
| Length of the vulva /mm | 77.94±1.9 | 68.86±2.18 | 0.002 |
| Length of the vulva ( Correct)/mm | 77.94±2.18 | 111.36±2.51 | 0.000 |
| Width of the vulva /mm | 40.31±0.79 | 38.81±0.91 | 0.218 |
| Width of the vulva (Correct)/mm | 40.31±1.03 | 62.76±1.19 | 0.000 |
| Vocalization of gilts | 0.17±0.07 | 0.89±0.08 | 0.000 |
| Climb across behavior | 0.05±0.07 | 0.95±0.08 | 0.000 |
